# Parent Versus Individual Cognitive Behavioral Therapy for Youth Anxiety: Protocol for an Overview of Systematic Reviews Over Time

**DOI:** 10.2196/48077

**Published:** 2023-06-29

**Authors:** Simon Byrne, Meg Richardson, Marcos Riba, Kana Imuta

**Affiliations:** 1 School of Psychology University of Queensland Brisbane Australia; 2 School of Psychology and Wellbeing University of Southern Queensland Ipswich Australia; 3 University of Queensland Library Brisbane Australia

**Keywords:** anxiety, cognitive behavioral therapy, systematic review, youth, overview, review

## Abstract

**Background:**

Cognitive behavioral therapy (CBT) has shown to be highly effective for treating youth anxiety; yet, there is ongoing debate as to whether involving parents improves outcomes. For example, parents who attend may learn CBT skills to help their child in an ongoing way; yet, they could also distract their child from treatment depending on how they interact. As evidence has accumulated, reviews and meta-analyses have attempted to examine the most effective treatment format. These reviews often have high impact in the field; however, they use varied methodologies and draw on different primary studies. Different formats of CBT for youth anxiety have been developed in relation to parental involvement, including youth-only CBT (Y-CBT; where the youth alone attends treatment), youth and parent or family CBT (F-CBT; where youths and their parents attend together), and, most recently, parent-only CBT (P-CBT; where the parent alone attends).

**Objective:**

This protocol describes an overview of systematic reviews comparing the relative efficacy of different formats of CBT for youth anxiety (Y-CBT, F-CBT, and P-CBT) over the study period. The protocol will also examine the moderating effects of variables on the efficacy of different formats; for example, youths’ age and long-term outcomes.

**Methods:**

We will analyze the results of systematic reviews that compare different levels and types of parental involvement in CBT for youth anxiety over the study period. A systematic review of medical and psychological databases (PsycINFO, PubMed, SCOPUS, Web of Science, Cochrane Library, and Embase) will identify reviews comparing the efficacy of different formats of parent involvement in CBT for youth anxiety. Data extraction will include (1) author names (and year of publication), (2) review design, (3) age range, (4) analysis type, (5) conclusions, and (6) moderators. This overview will present the relative efficacy of formats chronologically in a table and then describe the main results longitudinally in a narrative summary. A Measurement Tool to Assess Systematic Reviews, 2nd Edition (AMSTAR 2), quality rating will be given to each review, and the amount of primary study overlap across reviews will be quantified.

**Results:**

The last search was conducted on July 1, 2022. The reviews were published between 2005 and 2022. We found a total of 3529 articles, of which we identified 25 for the final analysis.

**Conclusions:**

This overview will compare and report the relative efficacy of Y-CBT, P-CBT, and F-CBT for youth anxiety over the study period, describe the heterogeneity across reviews and primary studies, and consider the moderating effect of relevant variables. It will describe the limitations of an overview, including the potential for nuance in the data to be lost, and provide conclusions and recommendations for conducting systematic reviews regarding parental involvement for CBT for youth anxiety.

**International Registered Report Identifier (IRRID):**

RR1-10.2196/48077

## Introduction

### Background

Cognitive behavioral therapy (CBT) represents a revolution for treating anxiety in adults and children. The efficacy of CBT for youth anxiety has been established in systematic reviews [1-3] and overviews [4,5]. During CBT, the therapist transfers skills to young clients so they can better manage their anxiety symptoms. As researchers have attempted to improve CBT protocols, they have examined whether it is advantageous to also involve parents in treatment. This approach seems logical, as parents and families are sometimes implicated in the maintenance of a child’s anxiety [6,7]. During CBT, parents can be coached in skills to manage their child’s anxiety and these skills can vicariously help the parent manage their own anxiety [3,8]. Yet, researchers have questioned the necessity of parental involvement in every intervention [2,7]. For example, a parent’s presence could increase the youth’s reliance on them and reduce their autonomy [9]. Barmish and Kendall [3] influentially stated, “we must resist the intuitive appeal to conclude that the inclusion of parents as active participants in CBT is preferable to child-focused CBT until the data provide the needed support for such a claim.” Whether parents enhance CBT for youth anxiety is one of the most frequently examined and contested topics in clinical psychology over the last 20 years.

Much of the debate regarding the merits of parental involvement in CBT has taken place in systematic reviews that have high impact in the field. These reviews provide a high-level analysis as they aggregate data from several primary studies. Yet, while these reviews are influential, they typically use different methodologies and only represent the accumulated evidence at a given time point. For example, the inclusion and exclusion criteria, types of studies available, and type of analysis conducted influences a systematic review’s outcomes and conclusions. Overviews (systematic reviews of systematic reviews) can potentially provide greater coverage over time and a higher level of analysis than an individual systematic review [10,11]. Understanding how different reviews summate and report on available evidence regarding treatment formats over time provides context as to how the field has developed. Furthermore, while early studies on this topic examined youth-only CBT (Y-CBT; where the youth alone attends treatment) versus youth and parent or family CBT (F-CBT; where the youth and parent attend treatment together), newer formats have examined parent-only CBT (P-CBT; where the parent alone attends treatment; [1,12]) so that parents can learn skills on how to manage their children’s anxiety. P-CBT can be advantageous when there is limited engagement with the youth or practical reasons prevent direct treatment. Moderating variables could also influence the efficacy of different treatment formats. For example, greater parental involvement may be influential for younger children [7], for longer-term outcomes [13], or when the parent is experiencing their own psychopathology [14].

This paper describes a novel protocol for an overview investigating the effects of parental involvement in CBT for youth anxiety over the study period. As results examine consistency and trends across individual reviews over time, we have permitted overlap (allowing primary studies in multiple reviews). As the reviews use varied methodologies and there is overlap, we displayed results as a narrative summary describing results for systematic reviews over time, rather than aggregating data (as in a meta-analysis). To our knowledge, this is the first overview to examine the effects of parental involvement in CBT for youth anxiety.

### Objectives

#### Primary Research Question

Our primary objective is to explore the relative efficacy of CBT for youths with anxiety by comparing Y-CBT, P-CBT, and F-CBT over the study period. The dependent variable is any quantitative indication of the youth’s anxiety (eg, recovery rates, youth- or parent-reported anxiety, treatment efficacy based on the level of evidence, etc).

#### Secondary Research Question

If the data permit, we will examine the moderating effect of variables including the youths’ age, parents’ psychopathology, and the long-term effects of Y-CBT, F-CBT, and P-CBT on youth anxiety. If any other theoretically interesting moderators are identified in the course of the literature review process, they may be included as well. This analysis will consider the effects of moderators on the relative efficacy of formats, and the results will be described in a narrative and interpreted in the discussion.

## Methods

### Protocol Registration

The protocol of this review is registered with the Open Science Framework [15].

### Search Strategy

The search will be undertaken by clinical psychologists who treat anxious youths (SB and M Richardson). The final set of articles will be developed in conjunction with a university librarian (M Riba). The search strategy involves searching databases for articles that have 4 general categories: “Cognitive Behavior Therapy” AND “Youths” AND “Anxiety” AND “Review.” Of the articles identified in this broad search, the screeners (M Richardson and SB) will hand-search articles related to “Parent/Family” treatments. We will also attempt to identify any additional eligible articles in a hand search. See Multimedia Appendix 1 for search queries in each database.

### Databases

The search will be conducted in the following electronic databases: PsycINFO, Web of Science, PubMed, Cochrane Library, SCOPUS, and Embase.

### Preferred Reporting Items for Systematic Reviews and Meta-Analyses

PRISMA (Preferred Reporting Items for Systematic Reviews and Meta-Analyses) guidelines have been followed in this systematic review. Data will be presented in a PRISMA flowchart.

### Inclusion and Exclusion Criteria

The inclusion and exclusion criteria for articles are summarized in Textbox 1.

**Inclusion and exclusion criteria for articles.**

**Inclusion criteria:**

- The article must state that it is a systematic review and involves a systematic search of medical or psychological databases (or both).
- The review must state that the treatment used is primarily cognitive behavioral therapy (CBT).
- The review must exclusively focus on the treatment of anxiety.
- The review should focus on treatment for a variety of anxiety disorders (mixed anxiety; >1 diagnoses) to reduce the potential differential effects of treatment on a particular anxiety disorder.
- The review must report a quantitative measure (eg, effect size, percent remission, etc) related to youth anxiety.
- Parental involvement in CBT can take any form (eg, cotherapist, psychoeducation, etc), as long as it is the focus of treatment.
- The review must discuss the relative efficacy of different formats for CBT; for example, it must make a direct comparison (eg, randomized controlled trials) or an indirect comparison (eg, comparison with to a control group) among the different formats of CBT for youth anxiety (youth-only CBT, youth and parent or family CBT, or parent-only CBT).
- The treatment must be intended for youths, so participants in reviews must be ≤21 years old.
- The treatment should be primarily face-to-face psychotherapy (ie, it should not be web-based or e-therapy).
- The included reviews must be in English-language peer-reviewed journals published from 2000 onward.

**Exclusion criteria:**

- Primary studies that are not systematic reviews.
- Reviews that focus on CBT and another concurrent treatment for the youth’s anxiety (eg, CBT and psychiatric medication). However, if a review includes primary studies where some participants are receiving a concurrent treatment, it will be included.
- Reviews should not focus on comorbidity between anxiety and another disorder as the target of treatment (eg, anxiety and autism or epilepsy); however, if a review involves primary studies where participants have comorbid conditions, it will be included.
- A review with only a narrative description of results and no analysis.
- In line with the most recent Diagnostic and Statistical Manual, 5th Edition [16], reviews that focus on obsessive compulsive disorder and posttraumatic stress disorder are excluded; however, if studies within the selected reviews include youths with these diagnoses among anxiety disorders, they will be included.

### Population, Intervention, Comparison, and Outcomes

The PICO is described as (1) population, comprising youths with anxiety; (2) intervention, involving P-CBT, Y-CBT, or F-CBT; (3) comparison, including other CBT formats different from the intervention (eg, P-CBT, Y-CBT, or F-CBT); and (4) outcome being the analysis of anxiety levels.

### Screening Stages

Two clinical psychologists (SB and M Richardson) will be involved in screening all articles with the following steps: (1) duplicates will be removed in EndNote (Clarivate), (2) articles will be transferred to Covidence for data screening and extraction, (3) articles will initially be screened based on the title and abstract by the 2 independent coders who are clinical psychologists, (4) the full text of the remaining articles will be screened by the 2 independent clinical psychologists to identify the final articles to be included, and (5) any discrepancies in screening decisions will be resolved through discussion between the 2 clinical psychologists.

### Data Extraction

The extracted data will include (1) author names (year of publication), (2) overall study design, (3) age range, (4) primary analysis type between different CBT formats (eg, controlled effect size where efficacy for a format is compared to another condition), and (5) conclusions regarding superiority among CBT formats. We may also code for other moderators such as youths’ age, parents’ psychopathology, long-term outcomes, and other variables identified through the literature review should there be adequate data.

### Management of Extracted Data and Synthesis

The coders (SB and KI) will extract the abovementioned variables for all included studies. To provide the most comprehensive overview, we shall describe the main results from systematic reviews with a variety of outcome measures over time. In this overview, we are interested in the relative performance of different formats of CBT for youth anxiety over the study period. Data extracted from this overview will initially be chronologically tabulated. The results will then be summarized in a narrative, based on the analysis type and outcome of each review (the results will not be presented as an overall meta-analysis, and results of individual reviews will be described over time). The analysis will include comparison among treatment formats. A primary analysis will examine the relative efficacy of P-CBT, Y-CBT, and F-CBT over time. A secondary analysis will be exploratory considering the effects of moderating variables, such as the youths’ age, parents’ psychopathology, and long-term outcomes, if the data permit. A general discussion will follow at the end of the review.

### Management of Overlap

In this overview, for the main analysis, we are interested in the relative performance of different CBT formats for youth anxiety over the study period. As individual systematic reviews were studied over time, overlap in primary studies across reviews is considered acceptable (ie, the same studies are included in multiple reviews published at different times). We are interested in how the accumulated data in each systematic review are reported over time.

In order to report the extent of overlap, we will include a citation matrix that visually displays the citation overlap across reviews [11,17,18]. We will also report the corrected covered area, which is a quantitative measure of primary study overlap across reviews [11,17,18]. We will also include some information or statistics about the degree of overlap across reviews (eg, the number of overlapping studies present in each review) [11].

### Quality Assessment

Each systematic review will be graded using the Measurement Tool to Assess Systematic Reviews, 2nd Edition (AMSTAR 2) [19]. It has 16 items and 7 critical domains: protocol registration, adequate literature search, justification of excluding studies, risk of bias of individual studies, appropriateness of meta-analysis, consideration of risk of bias when interpreting results, and assessment of publication bias. We will report the proportion of studies that meet each critical domain. For the purposes of this study, the AMSTAR 2 rating was calculated as high (none or 1 noncritical weakness), moderate (more than 1 noncritical weakness), or low (1 or more critical weaknesses). Uncertainty in quality ratings will be resolved through discussion between the 2 clinical psychologists (SB and M Richardson).

## Results

The last search was conducted on July 1, 2022. We identified 3529 articles across the search, which yielded 2189 unique articles. We found 25 systematic reviews that meet our criteria and compare CBT formats.

## Discussion

### Expected Findings

Youth anxiety is common and disruptive; however, CBT is a highly effective treatment for these conditions. Yet, there is ongoing debate regarding the most effective way to deliver CBT [3]. Parental involvement for youth anxiety has often been the norm; however, evidence for this approach has been mixed [2,8]. There are several systematic reviews examining parental involvement; yet, the majority of these are inconclusive [3]. This paper presents a novel protocol to answer this question by examining the results and conclusions of systematic reviews over time, rather than examining primary studies that are subject to heterogeneity. This overview examines trends and consistency in the efficacy of CBT formats as reported by reviews over time. This provides a high-level view of the topic by drawing on several systematic reviews over time. This overview will also consider potentially important moderators including youth’s age, long-term outcomes, and parents’ psychopathology.

### Strengths and Limitations

The strengths of this overview are that it will be conducted as a complex search across several medical and psychological databases. We will also take stringent measures of quality using AMSTAR 2, involve experienced child anxiety clinicians for coding, and analyze study overlap across reviews. The inclusion criteria also allow for a broad search across reviews with varying methodologies in order to maximize the search. A potential limitation of this overview approach is that nuance in the data may be lost due to our focus exclusively on systematic reviews rather than primary studies; for example, recent primary studies may not be included in this analysis as they are not included in systematic reviews. Furthermore, this study only considers efficacy, such that other important variables including cost, acceptability, and impact on functioning are not analyzed. Finally, while trends and directions in effects described in this overview are informative, they do not imply statistical significance.

### Conclusions

The results of this overview will inform clinicians about the most effective way to conduct anxiety interventions with youths. To date, clinical decisions regarding parental involvement are often based on mixed evidence and clinical intuition. This study will provide insight regarding how to tailor CBT for youths with anxiety by varying parental involvement depending on factors including the child’s age. This overview will also provide recommendations for conducting and reporting systematic reviews regarding parental involvement for CBT for youth anxiety. The overview methodology over time may help to examine other clinical questions, where there is high heterogeneity across primary studies. The results of this novel overview will be widely disseminated and they are expected to influence clinical practice.

## Appendix

Multimedia Appendix 1 Search queries for systematic reviews.

## Abbreviations

AMSTAR 2: Measurement Tool to Assess Systematic Reviews, 2nd Edition
CBT: cognitive behavioral therapy
F-CBT: youth and parent or family cognitive behavioral therapy
P-CBT: parent-only cognitive behavioral therapy
PICO: population, intervention, comparison, and outcomes
PRISMA: Preferred Reporting Items for Systematic Reviews and Meta-Analyses
Y-CBT: youth-only cognitive behavioral therapy

## Data Availability

All relevant data will be made available once the overview is complete.
